# Evaluation and enhancement of standard equations for renal function estimation in individuals with components of metabolic disease

**DOI:** 10.1186/s12882-021-02588-4

**Published:** 2021-11-22

**Authors:** Luigi Brunetti, Hyunmoon Back, Sijia Yu, Urma Jalil, Leonid Kagan

**Affiliations:** 1 Department of Pharmacy Practice; Ernest Mario School of Pharmacy; Rutgers, The State University of New Jersey, Piscataway, NJ USA; 2 Department of Pharmaceutics; Ernest Mario School of Pharmacy; Rutgers, The State University of New Jersey, Piscataway, NJ USA; 3 Center of Excellence in Pharmaceutical Translational Research and Education; Ernest Mario School of Pharmacy; Rutgers, The State University of New Jersey, Piscataway, NJ USA; 4 Lake Erie College of Osteopathic Medicine, Erie, PA USA

**Keywords:** Cockcroft-Gault, Renal function, Metabolic disease, Serum creatinine, Glomerular filtration rate

## Abstract

**Background:**

The primary objective of this study aims to test patient factors, with a focus on cardiometabolic disease, influencing the performance of the Cockcroft-Gault equation in estimating glomerular filtration rate.

**Methods:**

A cohort study was performed using data from adult patients with both a 24-h urine creatinine collection and a serum creatinine available. Creatinine clearance was calculated for each patient using the Cockcroft-Gault, Modified Diet in Renal Disease, and Chronic Kidney Disease Epidemiology Collaboration equations and estimates were compared to the measured 24-h urine creatinine clearance. In addition, new prediction equations were developed.

**Results:**

In the overall study population (*n* = 484), 44.2% of patients were obese, 44.0% had diabetes, and 30.8% had dyslipidemia. A multivariable model which incorporating patient characteristics performed the best in terms of correlation to measured 24-h urine creatinine clearance, accuracy, and error. The modified Cockcroft-Gault equation using lean body weight performed best in the overall population, the obese subgroup, and the dyslipidemia subgroup in terms of strength of correlation, mean bias, and accuracy.

**Conclusions:**

Regardless of strategy used to calculate creatinine clearance, residual error was present suggesting novel methods for estimating glomerular filtration rate are urgently needed.

## Introduction

Creatinine clearance (CrCl) is used to estimate glomerular filtration rate (GFR) to assess renal function. Numerous medications require dosage adjustment in the setting of reduced GFR and CrCl is often used to guide dosing of medications cleared by the kidney. The most accurate and clinically feasible estimate of CrCl is the measured 24-h urine CrCl; however, this method is not often practical and is time consuming. The Cockcroft-Gault equation published in 1976, [1] is the most commonly used formula to calculate CrCl using serum creatinine in the clinical setting. This equation was derived from a primarily Caucasian male population aged 18–92 years. While simple and in clinical use for more than 4 decades, there are several limitations one must acknowledge with the use of this formula. First, the population in 1976 was very different from the present-day population where over one-third of US adults are considered overweight or obese [2]. Further, in 2015, an estimated 30 million Americans were diagnosed with type 2 diabetes mellitus and continued increases in prevalence are expected [3, 4]. Both animal and human studies provide evidence that atherogenic lipid profile influences glomerular sclerosis and renal dysfunction, respectively, making dyslipidemia a relevant consideration in evaluating renal function estimates [5, 6]. Both obesity and diabetes are associated with altered muscle mass, [7, 8] which may influence serum creatinine a key variable in the Cockcroft-Gault equation. Another consideration is that the population used to derive the equation was primarily male (96%) and the extrapolation of the equation to females was based on estimates rather than objective data. Finally, the relatively small sample included in the study did not allow for subgroup analysis to determine in what populations calculated CrCl was not accurate.

The appropriate assessment of renal function is critical for drug dosing. More than half of all medications are cleared by the kidneys and inappropriate dose adjustment to account for potential drug accumulation may lead to drug toxicity. Therefore, an adept understanding of the accuracy, reliability, and nuances of the Cockcroft-Gault equation is necessary. Moreover, strategies to correctly classify the degree of renal dysfunction are likely to improve patient outcomes. The reliability and application of the Cockcroft-Gault equation (see equation below)

$$\frac{\left(140- age\ in\ years\right)\ast total\ body\ weight\ in\ kg\ }{72\ast sCr\ \left( mg/ dL\right)}\ast 0.85\ \mathrm{if}\ \mathrm{female}$$

in clinical practice may be influenced by several factors. First, the impact of weight must be considered. The physiological max GFR is approximately 120 mL/min; however, if the total body weight of an obese patient is entered into the equation the resultant value for CrCl often exceeds this threshold [9–11]. The question arises as to whether we should use ideal, lean, adjusted, or total body weight; a question that has been frequently tested with different answers [9–14]. The original Cockcroft-Gault equation suggested total body weight – but this was before the worldwide obesity epidemic. Second, serum creatinine may be influenced by malnutrition, cachexia, liver disease, and other conditions leading to lower muscle mass [13, 15, 16]. In clinical practice, serum creatinine is often below 1.0 mg/dL in these populations; therefore, GFR tends to be overestimated by the Cockcroft-Gault equation. Some have suggested rounding serum creatinine to 0.8 mg/dL or 1 mg/dL to account for this concern [17]. There is no substantial evidence to support these suggestions [17]. Overall, the aforementioned limitations are evident in individuals with components of cardiometabolic disease (i.e., obesity and diabetes). These individuals have altered body composition and as such estimates of renal function may less accurate. The primary objective of this study aims to test patient factors, with a focus on cardiometabolic disease, influencing the performance of the Cockcroft-Gault equation in estimating GFR. The secondary objectives were to determine if the development of a new CrCl equation by either modification of the original Cockcroft-Gault equation or using nonlinear regression incorporating disease states and race improved estimation relative to measured 24-h urine CrCl.

## Methods

### Data source and patient selection

A retrospective cohort study was performed using data extracted from the electronic discharge database and medical records at community medical center between January 2009 and July 2019. All consecutive adult patients (aged 18 years or older) with both a 24-h urine creatinine collection and a serum creatinine obtained within 24 h of each other were screened for inclusion. The institutional standard for measurement of creatinine is the Jaffe method. Only patients with comorbidities recorded in the electronic health record were further considered for inclusion. Patients were required to have a stable serum creatinine defined as less than a 20% fluctuation between two serum creatinine values measured within 48 h. Patients who were pregnant, had undergone amputations, in acute renal failure, or those on hemodialysis were excluded. Individuals with a serum creatinine greater than 2.5 mg/dL were excluded from the analysis since this would suggest Stage 5 Chronic Kidney Disease and previous data reported inaccuracies in estimation of CrCl using traditional equations in this population [18, 19]. Patients with a serum creatinine < 0.6 mg/dL were excluded since low serum creatinine may result in significant overestimates of renal function. Individuals with a measured 24-h urine CrCl below 10 mL/min were excluded since it would be expected that those in this group would receive dialysis [20].

### Data extraction and collection

All data were extracted from the patient discharge database and electronic health records (Cerner Millennium and Allscripts, Sunrise Clinical Manager). Patient height, weight, age, sex, race, and laboratory data were extracted from the medical record. Patient comorbidities were identified using International Classification of Diseases, 9th Revision or Clinical Modification or International Classification of Diseases, 10th Revision, Clinical Modification codes depending on availability. Once data were extracted, lean body weight, ideal body weight, and adjusted body weight were calculated based on standard equations [21–27]. Subsequently, CrCl was calculated using the Cockcroft-Gault equation. Various weight descriptors were then used for different versions of the Cockcroft-Gault equation. Some authors suggest rounding the sCr to 1.0 mg/dL if less than 1.0 mg/dL, especially in patients > 65 years of age. Therefore, calculated CrCl with the original Cockcroft-Gault equation was computed using sCr rounded to 1.0 mg/dL if less than 1.0 mg/dL. CrCl was also computed using the Modified Diet in Renal Disease (MDRD) and Chronic Kidney Disease Epidemiology Collaboration (CKD-EPI) equations other commonly used equations for estimating renal function [28–30]. All the standard equations used to estimate GFR are summarized in Table 1 [1, 28, 29, 31].

**Table 1:** Methods for calculating or measuring creatinine clearance

Method	Equation
Cockcroft-Gault	$\frac{\left(140- age\ in\ years\right)\ast weight\ in\ kg}{72\ast sCr\ \left(mg\left/dL\right.\right)}$ Multiply by 0.85 if female Original equation used total body weight
CKD-EPI	$141\ast \min \left(\frac{sCr}{k},1\right)\alpha \ast \max {\left(\frac{sCr}{k},1\right)}^{-1.209}\ast {0.933}^{age}\ast {1.018}^{if\ female}\ast {1.159}^{if\ African\ American}$ Where: sCr is serum creatinine in mg/dL k is 0.7 for females and 0.9 for males α is − 0.329 for females and − 0.411 for males min indicates the minimum of sCr/k or 1 max indicates the maximum of sCr/k or 1
MDRD	175 ∗ *sCr*^−1.54^ ∗ *Age*^−.203^ ∗ 0.742 (*if female*) ∗ 1.212 *if African American*
24-h urine CrCl	$\frac{Urinary\ creatinine\ \left(\frac{mg}{dL}\right)\ast urine\ volume\ (L)\ast 1000}{sCr\ \left(\frac{mg}{dL}\right)\ast 1440\ \left(\frac{\mathit{\min}}{day}\right)\ }$

### New equation development

Measured 24-h urine CrCl was used as the reference value to construct a prediction equation. All the equations derived for the prediction of CrCl were estimated using nonlinear regression. To consider sex effect of equation performance, the coefficient values for each male and female were estimated. Further, the effect of disease status on the equation performance was tested and selected based on the calculated *p* value from nonlinear regression. To develop a new CrCl equation, two strategies were considered. First, we considered modification of the Cockcroft-Gault equation. The coefficient values in the original Cockcroft-Gault eq. (72, 140, and 0.85 for women) were re-estimated using total body weight or lean body weight for each sex and then effect of disease state (obesity, diabetes, and dyslipidemia) was included.

We also considered substitution of various estimates of lean body weight in the Cockcroft-Gault equation. While the James equation for lean body weight estimation is commonly used due to its brevity, the Hume equation has been suggested as the optimal choice in special populations including obese patients (those with a body mass index [BMI] index above 37 kg/m^2^) [32]. We placed preference on this equation; however, tested others through construction and visual inspection of surface area plots and the influence this weight descriptor had on the performance of the Cockcroft-Gault equation versus measured 24-h urine CrCl.

Second, we performed multivariate regression analysis. For multivariate eq. 1, measured 24-h urine CrCl was divided by body surface area and then coefficient values for serum creatinine, age, and sex were estimated. For multivariate eqs. 2, 3, and 4 total, adjusted, or lean body weight were included with coefficient values to develop equation, respectively, as well as serum creatinine, age, and sex. After estimating coefficient values from base equations, disease states (obese, diabetes, and dyslipidemia) and race (white or non-white) were tested and included if *p* < 0.05 in the regression analysis. Only disease states present in at least 15% of patients in the analytic dataset were considered for evaluation in the regression.

### Statistical analysis

All results were summarized using descriptive statistics. Mean and standard deviation were reported for normally distributed continuous data and median and range were reported for data that were not normally distributed. Normality of data was assessed using visual inspection of histograms and the Shapiro–Wilk test. Binary data were reported as counts and proportions. The Pearson’s correlation coefficient between each of the calculated CrCl values and the measured 24-h urine CrCl and corresponding 95% confidence intervals were calculated using Fishers Z methods. The mean bias between the measured 24-h CrCl and various methods for calculated CrCl was defined as the difference between the two values and the precision was described using the 95% confidence interval for the difference. Root mean square error (RMSE) was calculated for each outcome in order to assess the degree of bias. Calculated CrCl not deviating more the 30% from the 24-h urine CrCl was considered to be accurate. This definition of accuracy was based on the original study validating the Cockcroft-Gault equation, which reported that Cockcroft-Gault calculated CrCl was within 30% of the 24-h urine CrCl [1] and others suggesting that if calculated CrCl is within 25% of measured 24 h urine CrCl it is considered accurate [18]. All analyses were conducted using SPSS, version 26 (IBM Corporation, Somers, NY) and R (R Core Team, Vienna, Austria).

## Results

**Fig. 1:**
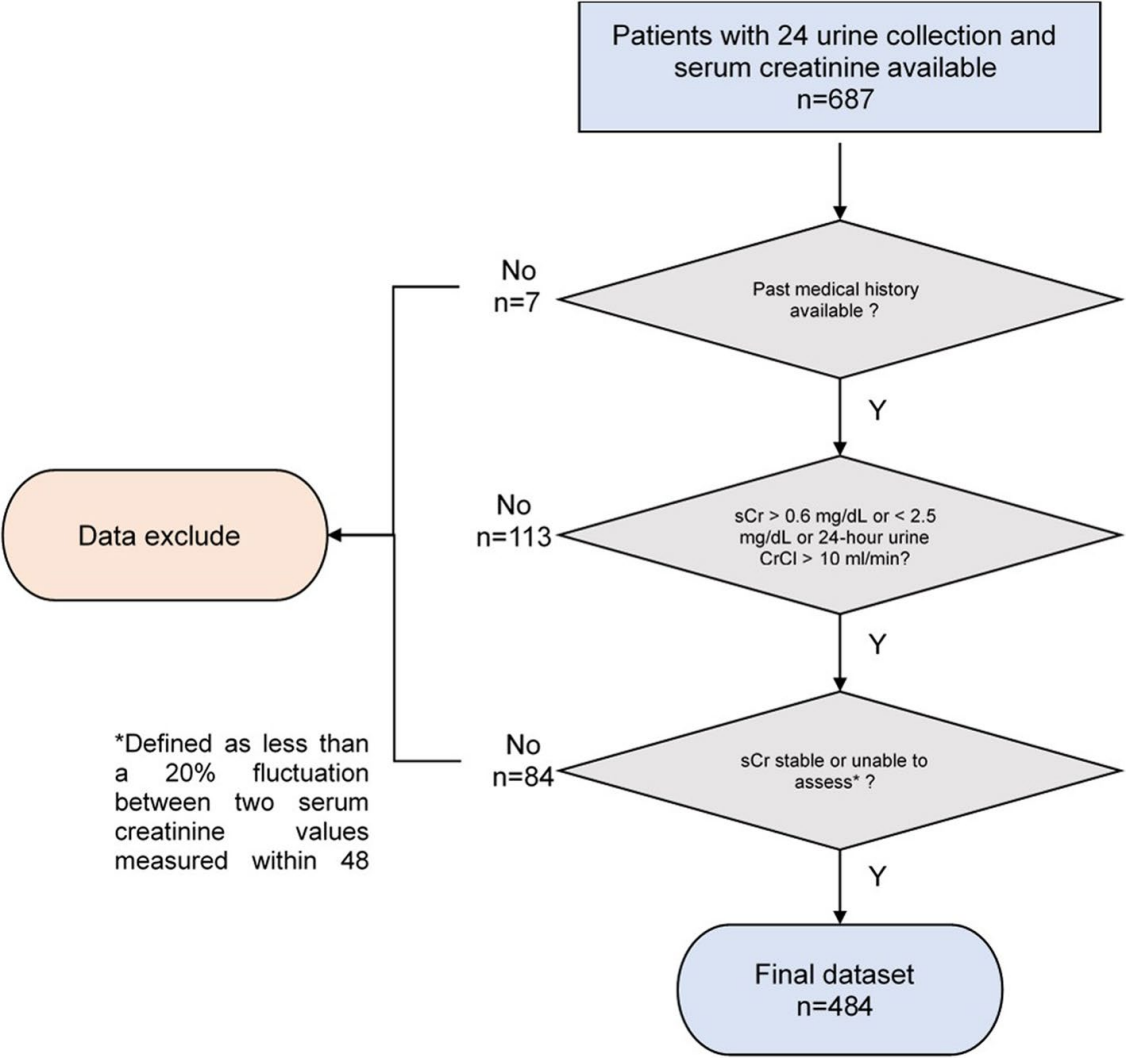
Data inclusion decision tree for evaluating various methods of calculating creatinine clearance

**Fig. 2:**
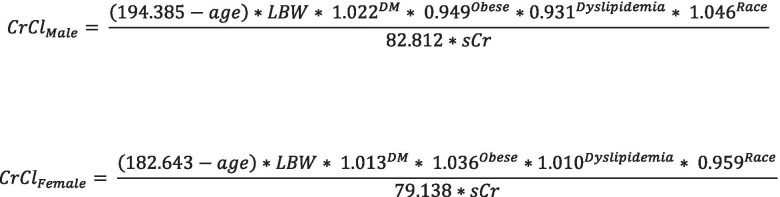
Modification of the Cockcroft-Gault equation to include select disease states and race

Figure 1 provides an overview of the patient selection process. After the initial screening, 687 patients were included in the dataset; however, upon application of the inclusion and exclusion criteria the final analytic dataset included 484 patients. Table 2 provides a summary of the patient characteristics. In the overall population, 44.2% of patients were obese, 44.0% had diabetes, and 30.8% had dyslipidemia. The mean calculated CrCl ranged from 55.8 ± 28.0 mL/min to 77.8 ± 37.2 mL/min depending on the method used. For comparison, the mean measured 24-h urine CrCl was 85.1 ± 47.5 mL/min. Depending on the subgroup (obese, diabetes, or dyslipidemia) there was variation in the mean calculated CrCl (Table 2). Using the traditional equations, adjusted and lean body weight produced the strongest correlation coefficients in the overall population and subgroups. Total body weight was similar and outperformed the other weight descriptors in terms of mean bias. Finally, adjusted body weight produced the greatest accuracy in most cases. Of the newly developed equations, multivariate (MVA)4 which incorporated lean body weight, select diseases, sex, and race performed the best in terms of correlation to measured 24-h urine CrCl, accuracy, and RMSE value (Table 3). This equation was further tested versus the other MVA equations. In addition, a modified Cockcroft-Gault equation was developed using the available data (Fig. 2). This equation incorporated obesity, diabetes, dyslipidemia, sex, and lean body weight. The correlation coefficient, mean bias, accuracy, and RMSE of each calculated CrCl method (including newly developed methods) versus the measured 24-h urine CrCl for the overall population and each subgroup is summarized in Table 4. The modified Cockcroft-Gault equation using lean body weight calculated using the Hume method performed best in the overall population, the obese subgroup, and the dyslipidemia subgroup in terms of strength of correlation, mean bias, and accuracy. The Cockcroft-Gault equation using adjusted body weight performed best in the diabetes subgroup.

**Table 2:** Comparison of patient characteristics and renal function estimates in the study population

Subject characteristic		Obese			Diabetes			Dyslipidemia		
	All Patients (n = 484)	None (***n*** = 270)	Yes (***n*** = 214	***p***-value*	None (***n*** = 271)	Yes (***n*** = 213)	***p***-value*	None (***n*** = 335)	Yes (***n*** = 149)	***p***-value*
Age (years, mean ± SD)	59.2 ± 17.8	60.0 ± 19.0	58.07 ± 16.2	0.23	60.2 ± 18.4	57.8 ± 17.0	0.14	58.1 ± 18.9	61.5 ± 14.9	0.03
Age greater than 65 years (n,%)	212 (43.8)	127 (47.0)	85 (39.7)	0.11	127 (47.0)	85 (40.0)	0.13	140 (41.8)	72 (48.3)	0.18
Female (n, %)	275 (56.8)	142 (52.6)	133 (62.1)	0.04	160 (59.0)	115 (54.0)	0.27	198 (59.1)	77 (51.7)	0.13
Race (n, %)				0.07			0.15			0.55
White Black Hispanic Asian Other	327 (67.6) 60 (12.4) 45 (9.3) 36 (7.4) 16 (3.3)	180 (66.7) 27 (10.0) 27 (10.0) 27 (10.0) 9 (3.3)	147 (68.7) 33 (15.4) 18 (8.4) 9 (4.2) 7 (3.3)		193 (71.2) 25 (9.2) 24 (8.9) 19 (7.0) 10 (3.7)	134 (62.9) 35 (16.4) 21 (9.9) 17 (8.0) 6 (2.8)		222 (66.3) 41 (12.2) 35 (10.4) 24 (7.2) 13 (3.9)	105 (70.5) 19 (12.8) 10 (6.7) 12 (8.1) 3 (2.0)	
Serum creatinine (mg/dL, mean ± SD)	1.1 ± 0.4	1.1 ± 0.4	1.1 ± 0.4	0.64	1.2 ± 0.4	1.1 ± 0.4	0.19	1.1 ± 0.4	1.2 ± 0.4	0.64
Weight (kg, mean ± SD)	86.0 ± 25.6	70.5 ± 14.0	105.6 ± 23.3	< 0.01	84.1 ± 24.8	88.5 ± 26.4	0.06	83.5 ± 24.3	91.7 ± 27.4	< 0.01
Body mass index (kg/m^2^, mean ± SD)	30.4 ± 8.1	24.8 ± 3.6	37.4 ± 6.7	< 0.01	30.3 ± 8.2	30.1 ± 8.1	0.85	29.8 ± 8.2	31.6 ± 8.0	0.03
Body surface area (m^2^, mean ± SD)	1.9 ± 0.3	1.8 ± 0.2	2.1 ± 0.3	< 0.01	1.9 ± 0.3	2.0 ± 0.3	< 0.01	1.9 ± 0.3	2.0 ± 0.3	< 0.01
Obese (n, %)	214 (44.2)	–	–	–	117 (43.2)	97 (45.5)	0.60	141 (42.1)	73 (49.0)	0.16
Heart failure (n, %)	53 (11.0)	22 (8.1)	31 (14.5)	0.03	7 (2.6)	46 (21.6)	< 0.01	17 (5.1)	36 (24.2)	< 0.01
Diabetes (n, %)	213 (44.0)	116 (43.0)	97 (45.3)	0.60	–	–	–	97 (29.0)	116 (77.9)	< 0.01
Dyslipidemia (n, %)	149 (30.8)	76 (28.1)	73 (34.1)	0.16	33 (12.2)	116 (54.5)	< 0.01	–	–	–
Measured CrCl_24-hr urine_ (mL/min, mean ± SD)	85.1 ± 47.5	77.8 ± 43.1	94.3 ± 51.2	< 0.01	81.3 ± 47.3	90.0 ± 47.5	0.05	85.1 ± 48.0	85.2 ± 46.6	0.98
CrCl CG_TBW_ (mL/min, mean ± SD)	89.3 ± 49.7	72.6 ± 33.7	110.4 ± 58.0	< 0.01	85.5 ± 50.2	94.1 ± 48.7	0.06	88.1 ± 49.1	91.9 ± 51.1	0.44
CrCl CG_IBW_ (mL/min, mean ± SD)	64.0 ± 31.5	64.6 ± 32.1	63.3 ± 30.8	0.65	60.6 ± 31.3	68.4 ± 31.3	< 0.01	64.4 ± 32.5	63.1 ± 29.2	0.66
CrCl CG_AdjBW_ (mL/min, mean ± SD)	74.1 ± 37.0	67.8 ± 32.2	82.2 ± 40.9	< 0.01	70.5 ± 37.1	78.7 ± 36.3	0.02	73.9 ± 37.2	74.6 ± 36.4	0.85
CrCl CG_LBW_ (mL/min, mean ± SD)	55.8 ± 28.0	50.9 ± 24.1	62.0 ± 31.2	< 0.01	53.1 ± 28.1	59.3 ± 27.6	0.02	55.6 ± 28.1	56.3 ± 27.8	0.81
CrCl CG_rounded sCr_ (mL/min, mean ± SD)	77.8 ± 37.2	62.9 ± 25.1	96.5 ± 41.4	< 0.01	74.1 ± 37.2	82.5 ± 36.8	0.01	76.3 ± 36.8	81.0 ± 38.0	0.21
CKD-EPI (mL/min, mean ± SD)	70.6 ± 28.7	71.3 ± 28.7	69.7 ± 28.8	0.55	68.3 ± 29.3	73.5 ± 27.7	0.05	71.4 ± 29.6	68.8 ± 26.7	0.35
MDRD (mL/min, mean ± SD)	71.8 ± 29.6	73.0 ± 29.6	70.5 ± 29.6	0.36	69.9 ± 30.6	74.3 ± 28.2	0.10	72.6 ± 30.5	70.3 ± 27.4	0.43

*p-value for comparison between group (i.e., no obesity versus obesity). Independent samples t-test for continuous data and chi-square for binary data

Abbreviations: *AdjBW* Adjusted body weight, *CG* Cockcroft-Gault equation, *CKD-EPI* Chronic Kidney Disease Epidemiology Collaboration, *CrCl* creatinine clearance, *IBW* ideal body weight, *MDRD* modified diet in renal disease, TBW = total body weight

**Table 3:** Development and testing of multivariate equations to calculate

Model	Equation	Correlation coefficient / RMSE
MVA 1	154.727 × *BSA* × *Scr*^−0.937^ × *age*^−0.301^ × 0.872^*Sex*^ × 0.983^*Race*^ × 1.042^*Diabetes*^ × 1.050^*Obesity*^ × 0.971^*Dyslipidemia*^	0.78 / 29.89
MVA 2	5.466 × *TBW*^0.976^ × *Scr*^−0.919^ × *age*^−0.359^ × 0.926^*Sex*^ × 1.003^*Race*^ × 1.034^*Diabetes*^ × 0.804^*Obs*^ × 0.957^*Dyslipidemia*^	0.79 / 29.25
MVA 3	0.985 × *AdjBW*^1.356^ × *Scr*^−0.924^ × *age*^−0.314^ × 1.014^*Sex*^ × 0.970^*Race*^ × 0.998^*Diabetes*^ × 0.924^*Obesity*^ × 0.964^*Dyslipidemia*^	0.80 / 28.65
MVA 4	1.431 × *LBW*^1.360^ × *Scr*^−0.920^ × *age*^−0.318^ × 1.043^*Sex*^ × 0.976^*Race*^ × 0.999^*Diabetes*^ × 0.913^*Obesity*^ × 0.963^*Dyslipidemia*^	0.80 / 28.65

Abbreviations: *AdjBW* adjusted body weight, *BSA* body surface area, *LBW* lean body weight calculated using the Hume equation, *MVA* multivariate, *TBW* total body weight. MVA 4 was selected for further development based on strength of correlation coefficient and RMSE value versus measured 24-h urine CrCl

**Table 4:** Correlation, bias, and accuracy between measured (24-h urine) glomerular filtration rate and estimated glomerular filtration rate is select populations

Method	Mean CrCl ± SD	Correlation coefficient (95% CI)	Mean bias (95% CI)	Accuracy within ± 30% n (%)	RMSE
***Overall (n = 484)***					
CrCl CG_ABW_	89.3 ± 49.7	0.77 (0.73–0.80)	−4.2 (− 7.2–1.2)	295 (61.0)	33.49
CrCl CG_IBW_	64.0 ± 31.5	0.72 (0.67–0.76)	21.1 (18.2–24.1)	224 (46.3)	39.13
CrCl CG_AdjBW_	74.1 ± 37.0	0.78 (0.74–0.81)	11.0 (8.3–13.6)	298 (61.6)	31.56
CrCl CG_LBW_	55.8 ± 28.0	0.78 (0.74–0.81)	29.3 (26.5–32.1)	162 (33.5)	42.72
CrCl CG_rounded sCr_	77.8 ± 37.2	0.73 (0.69–0.77)	7.3 (4.4–10.3)	289 (59.7)	62.26
CKD-EPI	70.6 ± 28.7	0.68 (0.63–0.73)	14.5 (11.4–17.6)	256 (52.9)	37.88
MDRD	71.8 ± 29.6	0.64 (0.58–0.69)	13.3 (10.0–16.5)	250 (51.7)	40.45
CrCl CG_modified_	85.4 ± 37.2	0.78 (0.74–0.81)	0.2 (− 2.4–2.9)	300 (62.0)	28.48
MVA	75.9 ± 26.9	0.77 (0.73–0.80)	4.2 (1.2–7.2)	282 (58.3)	28.65
***Obesity (n = 214)***					
CrCl CG_ABW_	110.4 ± 58.0	0.78 (0.72–0.83)	− 16.1 (− 21.0 – − 11.2)	129 (60.3)	39.89
CrCl CG_IBW_	63.3 ± 30.8	0.76 (0.70–0.81)	31.0 (26.4–35.6)	75 (35.0)	46.06
CrCl CG_AdjBW_	82.2 ± 40.9	0.79 (0.73–0.84)	12.2 (7.9–16.4)	131 (61.21)	33.69
CrCl CG_LBW_	62.0 ± 31.2	0.78 (0.72–0.83)	32.3 (27.9–36.8)	71 (33.2)	46.21
CrCl CG_rounded sCr_	96.5 ± 41.4	0.76 (0.70–0.81)	−2.2 (− 6.7–2.3)	135 (63.1)	67.24
CKD-EPI	69.7 ± 28.8	0.72 (0.65–0.78)	24.6 (19.7–29.5)	102 (47.7)	44.04
MDRD	70.5 ± 29.6	0.67 (0.59–0.74)	23.9 (18.7–29.1)	102 (47.7)	47.68
CrCl CG_modified_	94.3 ± 41.4	0.79 (0.73–0.84)	0.01 (− 4.2–4.2)	137 (64.0)	30.38
MVA	83.7 ± 30.1	0.77 (0.71–0.82)	16.1 (11.2–21.0)	128 (59.8)	30.59
***Diabetes (n = 213)***					
CrCl CG_ABW_	94.1 ± 48.7	0.73 (0.66–0.79)	−4.1 (− 8.9–0.7)	121 (56.9)	35.75
CrCl CG_IBW_	68.4 ± 31.3	0.73 (0.66–0.79)	21.6 (17.2–26.0)	101 (47.4)	39.02
CrCl CG_AdjBW_	78.7 ± 36.3	0.77 (0.71–0.82)	11.3 (7.2–15.4)	131 (61.5)	32.36
CrCl CG_LBW_	59.3 ± 27.6	0.76 (0.70–0.81)	30.7 (26.4–35.0))	80 (37.6)	44.21
CrCl CG_rounded sCr_	82.5 ± 36.8	0.70 (0.62–0.76	7.5 (2.9–12.1)	124 (58.2)	61.57
CKD-EPI	73.5 ± 27.7	0.66 (0.58–0.73)	16.5 (11.7–21.3)	101 (47.4)	39.29
MDRD	74.3 ± 28.2	0.61 (0.52–0.69)	15.7 (10.6–20.7)	100 (46.9)	42.62
CrCl CG_modified_	90.2 ± 37.4	0.76 (0.70–0.81)	0.1 (−4.0–4.3)	129 (60.6)	28.96
MVA	80.2 ± 27.4	0.77 (0.71–0.82)	4.1 (− 0.7–8.9)	128 (60.1)	29.71
***Dyslipidemia (n = 149)***					
CrCl CG_TBW_	91.9 ± 51.1	0.75 (0.67–0.81)	−6.7 (− 12.3 – − 1.1)	91 (61.1)	35.07
CrCl CG_IBW_	63.1 ± 29.2	0.75 (0.67–0.81)	22.2 (17.1–27.2)	72 (48.3)	38.29
CrCl CG_AdjBW_	74.6 ± 36.4	0.78 (0.71–0.84)	10.6 (5.9–15.4)	95 (63.8)	30.76
CrCl CG_LBW_	56.3 ± 27.8	0.78 (0.71–0.84)	29.0 (24.0–33.9)	55 (36.9)	41.97
CrCl CG_rounded sCr_	81.0 ± 38.0	0.72 (0.63–0.79)	4.3 (− 1.0–9.6)	95 (63.8)	58.70
CKD-EPI	68.8 ± 26.7	0.67 (0.57–0.75)	16.5 (10.8–22.1)	76 (51.0)	38.52
MDRD	70.3 ± 27.4	0.62 (0.51–0.71)	15.0 (9.0–20.9)	76 (51.0)	41.29
CrCl CG_modified_	84.6 ± 37.5	0.79 (0.72–0.84)	−0.6 (− 5.3–4.0)	101(67.8)	27.70
MVA	76.5 ± 27.8	0.78 (0.71–0.84)	6.7 (1.1)	98 (65.8)	27.22

Abbreviations: *AdjBW* adjusted body weight, *CG* Cockcroft-Gault, *CrCl* creatinine clearance, *LBW* lean body weight, *RMSE* root mean square error, *TBW* total body weight

## Discussion

The estimation of GFR using calculated CrCl is critical to select optimal medication dosing regimens. Individuals with obesity, diabetes, and dyslipidemia often are on many medications and have altered kidney function making calculating CrCl more critical. The overall purposes of this study were to identify the influence of cardiometabolic disease on GFR estimation and to enhance and develop CrCl equations. The current study identified several limitations with current practices in the calculation of CrCl. First, the Cockcroft-Gault equation uses the total body weight in calculating CrCl. There has been conflicting data regarding substituting adjusted body weight into the equation. We provide evidence that adjusted body weight is a reasonable consideration. Using this weight produced similar and, in some situations, better correlation, lower bias, and improved accuracy. Moreover, we demonstrate that the inclusion of obesity, diabetes, dyslipidemia, and race into a modified Cockcroft-Gault equation improves performance relative to the original Cockcroft-gault equation.

Previous studies have investigated the precision, bias, and accuracy or overall performance of GFR estimation using calculated CrCl in a variety of populations [11, 12, 16, 17, 31, 33, 34]. Our study is the first to consider all the components of cardiometabolic syndrome. The implications are significant in that the number of individuals with diabetes and obesity has continued to rise in the US and worldwide [2, 4, 35, 36]. Moreover, many early equations were developed in primarily Caucasian populations, leaving for debate whether the accurately capture the population as a whole. As such, we should ensure that validated equations still apply to the current population.

Some limitations to our study must be considered. First, 24-h CrCl was used as the reference value, an alternate to the gold standard. Ideally, injection of inulin and capturing its clearance by the kidneys would be preferable; however, this approach is seldom applied in the clinical setting due to its invasiveness, cost, time commitment, and lack of availability in retrospective data [37]. There are potential sources of bias when using 24-h CrCl as the reference value including errors in urine collection, increased creatinine secretion, and increased extrarenal degradation [38]. For example up to 20% of creatinine is not cleared through the kidney but rather through active secretion. This inherent bias will be present in any method using sCr in its estimation of GFR. Nonetheless, this strategy represents the most accurate clinically used method for measuring CrCl as an estimate of GFR. Second, we excluded patients with very high (> 2.5 mg/dL) or low (0.6 mg/dL) serum creatinine and those with a 24-h urine CrCl < 10 mL/min; therefore, extrapolating our results to these populations may not be appropriate. Regardless, drug dosing in patients in these extremes should be based on clinical context rather than calculated CrCl alone. The population age in this study was between (18 to 94 years) and included a large proportion of patients over the age of 65 years (43.8%). While this limits the external application to a younger population, advanced age represents a special population at an increased risk of drug toxicity. Renal impairment is frequently reported in older patients experiencing drug related iatrogenesis and improved assessment of renal function in this population is highly relevant. Moreover, hospitalized patients are often of advanced age and this population is more likely to have reduced CrCl requiring dosage adjustment. Finally, as with any retrospective study there is potential for information bias. Despite these limitations our findings provide important information for the clinician. Total body weight is the appropriate body weight descriptor to use in the Cockcroft-Gault equation to calculate CrCl. Using adjusted body weight is reasonable in individuals with obesity but provides modest benefit. Our modified Cockcroft-Gault equation using lean body weight outperforms all current methods and warrants further evaluation and validation.

Given that half of all medications or their metabolites are cleared by the kidney and roughly 3 billion prescriptions for medications are written annually many individuals may be at risk for underdosing (or overdosing) if renal function isn’t appropriately assessed [39, 40]. While renal dysfunction places patients at risk for adverse events when dosing is not appropriately adjusted, [41] dosage reductions when not necessary may increase risk of treatment failure which is especially concerning with antibiotics or chemotherapeutic agents [42]. Moreover, inaccurate assessment of renal function may influence patient selection in clinical trials [43]. Underestimation of CrCl may therefore exclude potential clinical trial candidates. Currently, FDA draft guidance for assessment of renal function in pharmacokinetic studies does not indicate a preference as to which formula is used to estimate kidney function [44]. Overall, clinicians should consider the patient population to determine the best strategy to assess renal function. In the clinical setting, determination of renal function to select a drug or drug dosing should not be done with the renal function estimate alone (i.e. Cockcroft-Gault), but rather an assessment of the clinical situation and repercussions for under or over dosing of the medication.

Further research is warranted to identify novel biomarkers to accurately estimate CrCl. Despite improvement of estimation using a modification of the Cockcroft-Gault or development of new equations using nonlinear regression to estimate CrCl, there remains residual error that cannot be explained. This error may be related to inherent limitations of using serum creatinine in the equations (extrarenal degradation and tubular secretion). Future studies should aim at identification of biomarkers that can accurately estimate renal function.

## Conclusions

Based on our study, total body weight is the appropriate weight descriptor to use in the original Cockcroft-Gault equation. Our modified Cockcroft-Gault equation using lean body weight outperforms other methods of calculating CrCl in terms of correlation, accuracy, mean bias, and RMSE value. Additional research is warranted to determine if this equation is correlated to drug exposure, toxicity, and efficacy.

## Data Availability

The datasets used during the current study are available from the corresponding author on reasonable request.

## Abbreviations

AdjBW: Adjusted body weight
BMI: Body mass index
CKD-EPI: Chronic Kidney Disease Epidemiology Collaboration
CrCl: Creatinine clearance
FDA: Food and drug administration
GFR: Glomerular filtration rate
LBW: lean body weight
MDRD: Modified Diet in Renal Disease
MVA: Multivariate
RMSE: Root mean square error
TBW: Total body weight
